# Exploring drivers of self-treatment with antibiotics in three agricultural communities of northern Tanzania

**DOI:** 10.1186/s13756-024-01453-x

**Published:** 2024-08-29

**Authors:** Kathrin Loosli, Fortunata Nasuwa, Matayo Melubo, Kunda Mnzava, Louise Matthews, Stephen E. Mshana, Blandina T. Mmbaga, Adrian Muwonge, Alicia Davis, Tiziana Lembo

**Affiliations:** 1 The Boyd Orr Centre for Population and Ecosystem Health, School of Biodiversity, One Health & Veterinary Medicine, College of Medical, Veterinary & Life Sciences, University of Glasgow, Glasgow, UK; 2 Kilimanjaro Clinical Research Institute, Kilimanjaro Christian Medical Centre, Moshi, Tanzania; 3 Department of Microbiology and Immunology, Catholic University of Health and Allied Sciences/Bugando Medical Centre, Mwanza, Tanzania; 4 Kilimanjaro Clinical Research Institute/Kilimanjaro Christian Medical University College, Kilimanjaro Christian Medical Centre, Moshi, Tanzania; 5 Digital One Health Laboratory, The Roslin Institute, The Royal (Dick) School of Veterinary Studies, University of Edinburgh, Easter Bush Campus, Edinburgh, UK; 6 School of Social and Political Sciences and School of Health and Wellbeing, University of Glasgow, Glasgow, UK

**Keywords:** AMR, One Health, Self-treatment, Antibiotics, Antibiotic use, Tanzania, Antimicrobial resistance

## Abstract

**Background:**

Antimicrobial resistance (AMR) poses a significant global health threat, particularly in low- and middle-income countries (LMICs). Self-treatment with antibiotics, the practice of using antibiotics without professional guidance, is often considered an important contributor to the emergence and spread of AMR.

**Method:**

This study investigated the drivers of self-treatment in three common types of agricultural communities in northern Tanzania. The research employed a comprehensive array of methods, including cross-sectional surveys (n = 790), interviews (n = 30) and observations (n = 178) targeting both antibiotic (human and animal) providers and users (patients and farmers). Qualitative interview data were analysed using a coding and association matrix, while descriptive analyses were performed on survey and observation data.

**Results:**

Self-treatment with antibiotics was highly prevalent in all communities. Between 41.0% (self-reported) and 60.3% (observed) of human antibiotics were obtained without a prescription and we observed that veterinary antibiotics were regularly purchased in retail shops without referral by a professional. Structural deficiencies in the healthcare system drove this practice: limited access to healthcare facilities, medication stockouts and prolonged waiting times were identified as key factors. The absence of safety nets like insurance schemes further contributed to self-medication. Retail shops offered a convenient and cost-effective alternative when antibiotics were inaccessible or unaffordable. Notably, informal networks comprising treatment vendors, friends or neighbours, as well as personal experiences played a crucial role in guiding individuals in their self-treatment decisions by providing advice on treatment choice and modalities.

**Conclusions:**

Addressing self-treatment requires a multi-faceted approach. Improving the availability and accessibility of antibiotics, enhancing healthcare services and involving retail vendors in antibiotic stewardship are essential. Structural issues like access to diagnostics and medicines must be tackled, alongside reducing barriers and incentivising individuals to use professional healthcare services. Training retail vendors to sell specific first-line antibiotics over the counter with guidance on appropriate usage should be considered. Such bottom-up interventions will enable sustainable promotion of responsible antibiotic use, mitigating AMR emergence and securing a healthier future for all.

## Background

The growing threat of antimicrobial resistance (AMR) poses a substantial challenge to global health, particularly in low- and middle-income countries (LMICs) [1, 2]. The health care systems of these countries are already burdened by a high prevalence of infectious diseases and therefore heavily rely on antimicrobial treatments, especially with antibiotics, in both the human and livestock sectors [3, 4]. A study by Klein et al. [4] revealed a rapid increase in antibiotic consumption by humans in LMICs from 2000 to 2015, with a rise of 114% (from 11.4 to 24.5 billion defined daily doses, DDDs). Moreover, the rates of antibiotic consumption surged by 77% (from 7.6 to 13.5 DDDs per 1000 inhabitants per year) and are projected to reach levels comparable to high-income countries (HICs) [4]. Similarly, increases in antibiotic consumption are reported in animals. Van Boeckel et al. [3] estimated a rise of global animal antibiotic consumption by 67% (from 63,151 to 105,596 tons) between 2010 and 2030, mostly driven by a transition from extensive subsistence farming to intensive large-scale farming systems in LMICs. Nevertheless, due to inadequate health coverage, patients and farmers often resort to other sources of antimicrobials without receiving proper professional guidance on their appropriate use [5].

The vulnerability of LMICs to the social and economic consequences of AMR is exacerbated by their fragile healthcare systems, resulting in higher mortality rates in humans and animals [1, 2, 6]. Projections indicate that by 2050, AMR could lead to 10 million deaths globally, with over four million of these occurring in sub-Saharan Africa [1]. In the absence of interventions, global production losses resulting from AMR could amount to a staggering $100 trillion between 2016 and 2050, with LMICs bearing a disproportionate burden due to their heavy reliance on agriculture as a vital component of their economies and livelihoods [1, 7]. Consequently, an urgent need exists for enhanced antimicrobial stewardship to address the suboptimal and inefficient use of antimicrobials in humans and animals. Given their importance, stewardship activities are a central and integral component of AMR national action plans in numerous countries within the region, including Tanzania which is the focus of our study [8]. By enhanced antimicrobial management, we can mitigate the risks associated with the development and spread of AMR, and work towards achieving the sustainable development goal of ensuring healthy lives and promoting wellbeing for everyone [9].

In LMICs, increasing levels of antibiotic consumption and the development of AMR are attributed to the higher availability and widespread over-the-counter sale of antibiotics through the retail sector [10–12]. In countries where public health system coverage is low, retail outlets are vital sources of lifesaving drugs [12–14]. These include both licensed and unlicensed drug shops. For example, in Tanzania, ‘type 1’ pharmacies, i.e. those led by a registered pharmacist and which are abundant in urban settings, are allowed to sell antibiotics [15]. On the contrary, most of the licensed drug shops found in rural areas, so called “Accredited Drug Dispensing Outlets” (ADDOs, see Rutta et al. [16]), can only sell a limited range of prescription drugs [15]. Yet, antibiotic sales through unlicensed outlets are widespread and tolerated by inspectors, particularly in the most remote settings, as it is recognised that restricting access to essential medicines would have worse consequences than their uninformed use [12, 17, 18]. In the animal health sector, veterinary medicines, including antibiotics, are often sold in agrovet shops, even though it is technically illegal for farmers to treat their own animals with antimicrobials [15, 19]. Other important informal sources of antibiotics in LMICs include friends or neighbours, traditional healers, community health workers, ambulant vendors or market sellers [12, 15], despite the fact that these are not officially licensed to dispense antibiotics [15].

Hand in hand with the over-the-counter sale of antimicrobials is the issue of self-treatment which is typically defined as the use of medicines, such as antibiotics, without prior diagnosis and counselling by a healthcare professional. Self-treatment is often associated with over- or sub-optimal use, for example using antibiotics when they are not the correct treatment (for instance, for viral or parasitic diseases), not adhering to the recommended duration or not observing withdrawal periods [5, 13, 20–22]. Self-treatment to tackle both human and animal health issues is widespread in sub-Saharan Africa [11, 12, 23], including Tanzania [14, 24–28]. While informative of wider ABU patterns, most research has not focused specifically on the drivers of self-treatment [23, 27] or has examined either healthcare users’ or providers’ perspectives in isolation [14, 18]. Methodologically, the depth of many of these studies has been constrained by the limited range of methods employed, for example, cross-sectional household surveys or interviews [12]. This is problematic, because health seeking practices are complex and influenced by economic, structural and social factors [21, 29–31]. For example, provider-user relationships and communication, and social networks shape where and when people seek treatment [32]. External factors such as poor living standards, low socio-economic status or the lack of infrastructure, including roads and water as well as health care infrastructure, limit people’s choices and agency over their own health decisions [21, 23, 29, 32]. To thoroughly investigate such complexities, a wide scope and a diverse range of research methods are required. This is a requisite to find sustainable and workable solutions to curb problematic practices associated with antibiotic self-treatment.

When studying self-treatment in sub-Saharan Africa, it is crucial to examine human and animal health provision jointly in line with a One Health approach, given their interconnectedness. One Health is an integrated, unifying approach that aims to sustainably balance and optimise the health of people, animals, and ecosystems, recognising that these areas are closely linked and should be addressed together to achieve optimal health outcomes. In agricultural communities, choices around livestock treatment are driven by the need to safeguard food production and livelihoods—animals are a source of savings and, in case of emergency, of instant cash [33]. Therefore, antibiotic treatment is often perceived as essential to maintain the health and productivity of this critical asset. Additionally, antibiotics are sometimes used as growth promoters to enhance livestock growth rates, although this practice has been shown to be relatively rare in small-scale farming communities [13, 34, 35]. Antimicrobial practices in livestock can also have implications for human health. For instance, consumption of animal products from livestock treated with antibiotics can result in spread of antibiotic-resistant bacteria and residues from animals to humans [36]. Local practices affecting transmission, such as use of water sources shared with livestock and non-standardised processing of milk, can also play a role [37]. In addition, crossover use of veterinary drugs in humans and vice versa has been reported [38]. For this reason, regulating a drug only in one sector might not curb its use in another.

In this study, we investigate self-treatment in humans and animals in three agricultural communities of northern Tanzania with the aim to obtain a comprehensive understanding of when, how and why self-treatment is practised in these and similar contexts. For the purpose of this study, we consider self-treatment as any instance when human or veterinary antibiotics are bought in retail shops without a prescription and are subsequently administered to a person or an animal by patients or farmers themselves. Specifically, we investigate the (1) occurrence of self-treatment, and include the most commonly used antibiotics for this practice; (2) drivers of self-treatment; and (3) sources of information used to guide self-treatment. We examine drivers of self-treatment according to a framework for access to healthcare outlined in earlier work [5, 39]. In brief, the framework captures variability of access to healthcare in low-resource settings according to five dimensions: availability, accessibility, affordability, adequacy and acceptability. These encompass key components of self-treatment, including health infrastructure and means to access it as well as service organisation, costs and factors that influence users’ satisfaction.

To gain a deeper understanding and effectively represent the dynamics involved in the multifaceted practice of self-treatment, we draw upon data generated through a wide array of research methods. These methods include cross-sectional surveys, interviews and direct overt observations targeting both health providers (both human and animal) and users. By employing this diverse range of research techniques, we aim to provide a comprehensive understanding of self-treatment practices and the contextual systemic factors that drive them. The use of various research methods allows us to acquire in-depth insights, enhancing the validity and robustness of our findings. Consequently, our results are well suited to inform evidence-based interventions and policies to address self-treatment effectively. Ultimately, our study can inform healthcare policies at both local and higher levels, facilitating the implementation of multifaceted interventions that improve access to healthcare.

## Methods

### Study sites

The study was conducted in three districts of northern Tanzania representative of three livestock production systems—pastoral (Ngorongoro District, Arusha Region), agropastoral (Misungwi District, Mwanza Region) and smallholder (Mwanza District, Kilimanjaro region)—that are predominant across Africa. These livelihood production systems are linked to particular environmental and social conditions [40]. Pastoral systems are found in semi-arid areas and rely on livestock production, utilising long-distance, seasonal movements of herds for grazing in response to variable rainfall patterns. Agropastoral systems mix farming-based agriculture and livestock production with varying levels of livestock mobility, including seasonal movements and communal grazing [41]. Smallholder systems have the fewest numbers of livestock per household alongside more intensive agricultural production [40]. The three study populations were selected based on representation of these production systems and on their differential access to referral hospitals and other health facilities. We hosted an initial introductory meeting at KCRI in Moshi to which District Medical Officers and District Veterinary Officers of the three study districts were invited. Together, they assessed the availability and accessibility of health services “on the ground” within the different communities in their individual districts. The focus was on examining the distribution of public facilities and presence or absence of public veterinary services, and on evaluating relative travel distances to the nearest district hospital. Two villages in each study district/region (representative of the three livestock production systems of focus), were then selected, one at proximal and one at distal locations relative to their regional referral hospitals. Comparability across regions was ensured based on the list of criteria for village selection created in the workshop, including relative (human and livestock) population size, number of sub-villages, and presence/absence of governmental, non-governmental or private health/veterinary services, drug shops and health facilities. However, due to large variations in these factors at district-level, such characteristics were not always perfectly comparable across districts.

### Characteristics of health providers included in the study

The following providers were included in some or all of the research activities included in this study:

1. *Public human health facilities*: Public human health facilities in the study area comprised government-run lower tier health services such as dispensaries and health centres. These facilities are staffed with medically trained nurses, and clinical and/or medical officers [15]. First- and second-line antibiotics are prescribed and dispensed in these facilities.
2. *Human health retail outlets*: Formally licensed private human health retail outlets in our study area included “Accredited Drug Dispensing Outlets” (ADDOs) (see Rutta et al. [16]). These outlets are permitted to dispense a limited range of antibiotics by prescription only and are staffed by individuals with a minimum of four years of training in human health, up to diploma or certificate level [15].
3. *Public veterinary services*: Governmental public veterinary services in the study areas are represented by the Livestock Field Officers (LFOs). LFOs provide on-demand animal health services to farmers. These services include diagnosis and treatment, vaccinations and artificial insemination, among others, with farmers paying for the LFO's visit and services [15].
4. *Animal health retail outlets*: Formally licensed private veterinary retail outlets are known as “agrovet stores” or “agrovets”. These stores sell a wide range of medicines, including antibiotics, which are intended to be administered under veterinary supervision only, despite being sold to farmers [31]. Agrovet staff receive short-course training [15] and may not have a formal animal health background [31].
5. *Informal providers*: Unlicensed informal providers in the study area encompassed open markets and ordinary shops where antibiotics, for both human and animal use, are sometimes illegally sold by untrained staff [15].

### Data sources

A range of data collection activities were conducted in the 6 villages as outlined below, resulting in four datasets. Target groups included patients and farmers as antibiotic users, as well as public and private antibiotic providers. A summary of data acquisition methods is given in Table 1. Some activities were only conducted in one village per district, due to time restrictions. The village was chosen depending on its accessibility and the availability of a range of health services, with villages with a higher diversity of health services being preferred. The resulting datasets were used for data triangulation as detailed below.

**Table 1:** List of activities that were carried out in three districts of northern Tanzania to generate data on self-treatment. Target participant group and sample size are described

Method	Village per district	Participant group	Sample size by district and provider type	Type of data acquired
Exit survey	2	Clients or patients/farmers visiting various human and veterinary health facilities	**Mwanga**: 287 clients/patients at:	Quantitative: survey answers
			2 public human health providers	
			3 human health retail providers	
			3 informal human health providers	
			11 clients/farmers at:	
			2 animal health retail outlets	
			**Misungwi**: 303 clients/patients at:	
			2 public human health providers	
			4 human health retail providers	
			18 clients/farmers at:	
			2 animal health retail outlets	
			1 informal animal health provider	
			**Ngorongoro**: 118 clients/patients at:	
			2 public human health providers	
			5 human health retail providers	
			53 clients/farmers at:	
			4 animal health retail outlets	
			**Total: 790 clients/patients/farmers at 30 health outlets**	
Drug bag interviews	1	Households	**Mwanga**: 10 households	Quantitative: survey answers Qualitative: interview transcripts
			**Misungwi**: 10 households	
			**Ngorongoro**: 10 households	
			**Total: 30 households**	
Provider surveys and observations	1	Human and veterinary antibiotic providers	All human and animal antibiotic dispensers in one village per district, including:	Quantitative: survey answers and observations recorded in a survey form Qualitative: fieldnotes for observations beyond survey form
			**Mwanga**: 23 observations at:	
			1 public human health providers	
			2 human health retail providers	
			1 animal health retail providers	
			**Misungwi**: 61 observations at:	
			1 public human health providers	
			3 human health retail providers	
			2 animal health retail providers	
			**Ngorongoro**: 94 observations at:	
			1 public human health providers	
			3 human health retail providers	
			3 animal health retail providers	
			**Total: 178 observations at 17 antibiotic providers:**	

#### Exit surveys

A short questionnaire (available here: https://researchdata.gla.ac.uk/id/eprint/1561) was administered to all clients visiting human (n = 21) and veterinary (n = 9) drug dispensing outlets (including private retail outlets, markets and public facilities) in all six villages. Each outlet was surveyed for one to three working days depending on size and opening hours. The digital questionnaire was designed using Light House Studio 9.6.1. All customers exiting drug dispensing outlets within one to three working day(s) were asked to participate by a local researcher from Kilimanjaro Clinical Research Institute (KCRI) who also administered the questionnaire in Swahili or Maa using tablet computers. This component of the study took place during January–August 2021. A research assistant asked the survey questions and entered the answers given by the participant into the survey form on the tablet in Swahili or English.

The survey included basic participant demographics and questions about the purpose of visiting the outlet. Specifically, we asked what medicines were bought and for what reason, if a prescription had been acquired beforehand, how well participants were able to access services in the outlet (including availability and cost of drugs as well as travel time to the outlet), how the quality of service was, and if and what type of advice was offered. Finally, participants were asked about their understanding of the terms “antibiotic” and “antibiotic resistance”. To ensure confidentiality, no names were asked of participants, and only aggregated data at the district level are presented in this article. Participation was based on informed consent that was obtained after providing participants with appropriate information about the study, including confidentiality procedures. All data were stored on encrypted and password-protected computers. Overall, 790 participants answered the survey, 708 at human health outlets and 82 at animal health outlets.

#### Drug bag

The “drug bag” method is a participatory social science method designed to produce accurate antibiotic use data and to provide a talking point for participants to discuss their experiences with antibiotic treatment [42]. Study participants are asked to examine physical samples of antibiotics available in their village to understand their knowledge of these and patterns of use. We purchased all human and veterinary antibiotics available in the three study villages, including different dosage forms and brands, from a variety of sources (public health facilities, drug shops and agrovets). The antibiotics were placed in a “drug bag” specific to the visited village, i.e. this contained all different formulations and brands we had found in that particular village. The drug bag was then presented to ten households in each district (30 households in total). Households were selected through convenience sampling and contacted through the (sub-) village chairperson and asked to participate in the study. Only households with livestock (poultry, cattle, sheep and/or goats) were chosen. The chairperson was asked to choose them from various parts of the village and, in every location, at least two sub-villages were visited. Sub-villages are administrative units that divide villages, with an average village having three to five sub-villages, each with their own leader/chairperson. The interviews were conducted by a KCRI research assistant in Swahili or Maa. Answers were entered in a questionnaire form (available here: https://researchdata.gla.ac.uk/id/eprint/1561) designed using Open Data Kit (ODK) which was then downloaded from Google Drive the same day and saved onto local hard disks. Each session was also voice recorded if participants agreed to it (n = 28) and extensive notes were taken by an observing field team member.

Participants were asked to sort the drugs in the bag into piles of “known” antibiotics according to two different categories, ‘antibiotics they had used before’ and ‘antibiotics they used often or regularly’. We then asked them questions about the three most used antibiotics in the household to find out what disease they were used for, where they had been accessed from, how they were administered and where the information about how to use them came from. Further, we asked participants to point out all antibiotics they were unable to obtain locally and then asked them why they thought they were inaccessible. Next, we enquired about antibiotics that they perceived to not be working well and the circumstances in which the lack of efficacy occurred. Finally, we asked about human antibiotics they had used to treat animals and veterinary antibiotics they had used to treat humans, and why they chose to do so. To ensure confidentiality, no names were asked of participants, and only aggregated data at the district level are presented in this article. All audio recordings and transcriptions were fully anonymised. Participation was based on informed consent that was obtained after providing participants with appropriate information about the study, including confidentiality procedures. All data were stored on encrypted and password-protected computers.

#### Provider surveys

In order to purchase the antibiotics needed for the drug bag component of the study, we visited all local formal and informal community providers of antibiotics in each of the three village (11 human providers and six veterinary providers in total). We collected information on the types of antibiotics sold at each source. In addition, a short questionnaire (available here: https://researchdata.gla.ac.uk/id/eprint/1561) was administered to providers in Swahili or Maa by a local research assistant. Answers were entered in a questionnaire form designed using ODK which was then downloaded from Google Drive the same day and saved onto local hard disks. Additionally, field notes were taken by an observing field team member.

This survey also asked about basic participant demographics as well as information about the locality and nature of the health facility and providers’ education and experience relating to health provision. We made an inventory of all antibiotics dispensed in each facility. Providers were asked to name their top three most dispensed antibiotics followed by questions about use and availability of each of these. We aimed to understand what diseases they were dispensed for and if they were readily accessible to users. We were specifically interested in ascertaining whether stockouts occurred, whether affordability was an issue and what dispensers would then sell in these situations. Furthermore, we asked if patients or clients needed a prescription to receive the antibiotics and if they usually presented one and from whom. We also recorded what kind of advice about antibiotic use providers considered important to give patients/clients, if and why they assessed specific antibiotics to be of good quality and what they would do in case the treatment failed to cure a client or patient. Another topic we discussed was the use of animal drugs in humans and vice versa to understand if providers were aware of such practices. Finally, we asked about the organisation of the facility, specifically how stocks are managed, how providers decide which antibiotics to stock, how they source antibiotics and if they are visited by pharmaceutical representatives. To ensure confidentiality, no names were asked of participants, and only aggregated data at the district level are presented in this article. Participation was based on informed consent that was obtained after providing participants with appropriate information about the study, including confidentiality procedures. All data were stored on encrypted and password-protected computers.

#### Observations

We also undertook observations in all of the aforementioned localities. Observations are frequently employed in social sciences to gather data by observing participants' behaviours, relationships or reactions, and by recording processes or events [43]. This method is particularly valuable in mixed-method studies for data triangulation, where findings from one data source or collection method are verified against those from another [43].

Our main goal was to understand prescribing, dispensing and counselling practices as well as aspects of patient-provider interactions. While we were not able to observe prescribing practices directly, we focused on observing whether patients presenting at outlets had a prescription, and noted the condition this prescription was associated with and whether this was followed by both retail providers and patients. Two research assistants stayed at each facility for a minimum of one and a maximum of two full working days. We undertook overt observations, with the observers seated next to the counter to be able to see and hear both providers as well as clients. The provider informed his/her clients about the observer and gave them the option to ask for exclusion from the study, which no client/patient did take.

During the observations, one assistant filled in a form (available here: https://researchdata.gla.ac.uk/id/eprint/1561) previously designed in ODK on a tablet computer for each client/patient/farmer, while another team member took handwritten notes. We recorded the time clients spent in the facility, and what drugs were bought and, if the information was available, for what condition/s. We noted whether a prescription was presented, the counselling and advice given regarding the drug of choice and/or their use, and in what form the drugs were dispensed (i.e. in a box, blister or loosely). No communication or action with patients/clients/farmers or providers that would interfere with the provider–client interaction was carried out, i.e. the research assistants did not ask any questions nor reacted to anything the client/patient or provider did or said. This approach is defined as ‘direct’ observation [43]. However, we did check back with the provider on occasion to ensure we had recorded all information correctly (like names of medicines or illnesses mentioned). To ensure confidentiality, no names were asked of participants, and only aggregated data at the district level are presented in this article. Participation was based on informed consent that was obtained after providing participants with appropriate information about the study, including oral confidentiality procedures. All data were stored on encrypted and password-protected computers.

### Data analysis

#### Quantitative data

Quantitative questionnaire data from exit and provider surveys, as well as observations and some drug bag answers recorded in a survey form, were available from the software as CSV files. These were imported into R (version 4.2.0, R core team [44]) for descriptive analyses using R packages contained in ‘tidyverse’.

#### Qualitative data

Drug bag interview recordings (n = 28) were transcribed from Swahili or Maa to English and the transcripts were analysed using NVivo 12 (version 12.7.0) [45]. Where participants had not agreed to be recorded (n = 2) field notes were coded instead. A coding network was developed based on a literature review [5] and earlier work in these study communities [22, 32]. The network was supplemented with nodes based on inductive coding. Regular cross-checking and discussion of codes amongst authors was done to ensure validity of the nodes. In addition, comparisons with the literature and survey datasets were made for consistency, concurrence and agreement. An association matrix between individual nodes was used to compare codes related to self-treatment with codes on barriers to healthcare. Similar comparisons were made between self-treatment and sources of information on treatment and drug use.

#### Data triangulation

The quantitative analysis yielded descriptive insights into the frequency of self-treatment and antibiotic usage practices, as well as the number of individuals seeking antibiotics or advice from different sources in each district. Observations validated self-reported data. However, the qualitative findings provided a deeper understanding of the underlying context and motivations behind these behaviours. By incorporating both quantitative and qualitative data, we were able to not only quantify actions and practices but also to offer explanations for why they occur [46]. This comprehensive approach allows for a more thorough and nuanced exploration of self-treatment with antibiotics, ultimately contributing to a more robust understanding of this significant public health issue.

#### Frameworks used for analysis

To categorise different antibiotic products, we used the World Health Organization (WHO)’s Access, Watch, Reserve (AWaRe) classification of antibiotics for human consumption [47] and the ABCD categories for veterinary antibiotics created by the European Medicine Agency (EMA), namely Avoid, Restrict, Caution and Prudence [48], to highlight the importance of each antibiotic for human health and its potential for resistance. Specifically, according to these categorisations, ‘Access’ and ‘class D’ antibiotics are first-line treatments for humans and animals which should be used widely, while ‘Watch’ and ‘class C’ antibiotics should be employed more selectively. ‘Reserve’ and ‘class B’ antibiotics should only be used as a last resort in case of resistant infections. ‘Not recommended’ or ‘class A’ antibiotics should not be administered, as they are not recommended and/or not approved for use at all.

Findings regarding systemic drivers of self-treatment are presented under five themes according to the framework for examining access to quality healthcare in low-and-middle income countries by Obrist et al. [39]: (1) availability relates to the physical existence of health facilities for patients to attend or of drugs or diagnostics for clients to buy and use; (2) accessibility is defined as the ability of patients to reach, attend and use the available health services; (3) affordability considers the ability of patients to pay for health services, including drugs and related costs like transport or loss of productivity during the time of health seeking; (4) adequacy is defined as the ability of the organisational structures and processes of the health providers to meet patients’ requirements, i.e. if opening times and timing align with patients’ daily schedules and if available infrastructure is well-kept and usable; and (5) acceptability relates to the appropriateness of the form of service provision as perceived by the patient [5, 39].

Below, our narrative is developed in line with One Health principles, and, therefore, integrates human and animal health data. The reasoning behind this is to highlight the inherent interconnectedness of processes around human and animal health in agricultural communities of East Africa as well as the constraints within which both healthcare sectors operate. These challenges often result in health-seeking behaviours that are common amongst community members tending to the health of their families and livestock.

## Results

### Summary of respondent characteristics

A summary of participant characteristics over all datasets is shown in Table 2. In all datasets at least 35.3% and a maximum of 64.7% of participants were female. While 82.4% of providers completed college, participants of the exit survey and drug bag mostly completed primary school (50.8% and 73.3%, respectively). In the exit survey, participants who had attained no education were found mainly in the pastoral study sites, whereas higher levels of education (secondary school) were more likely in smallholder settings. Regarding age, most exit survey participants were between 20 and 30 years old (39.1%), while 30.0% of primary respondents in the drug bag dataset were over 60 years old. Overall, pastoralists were slightly younger and smallholders slightly older in both the exit survey and the drug bag dataset.

**Table 2:** Socio-demographics of participants in exit survey, provider survey, observations and drug bag interviews for each study districts and overall

Data sets	Factors	Level	Agropastoral district		Smallholder district		Pastoral district		Total districts	
			Count	%	Count	%	Count	%	Count	%
Exit survey		Total participants	n = 321		n = 298		n = 171		n = 790	
	Gender	Female	217	67.6	147	49.3	63	36.8	427	54.1
		Male	104	32.4	151	50.7	108	63.2	363	45.9
	Education	None	65	20.2	7	2.3	70	40.9	142	18.0
		Some Primary School	24	7.5	31	10.4	17	9.9	72	9.1
		Completed Primary School	187	58.3	164	55.0	50	29.2	401	50.8
		Some Secondary School	17	5.3	22	7.4	13	7.6	52	6.6
		Completed Secondary School	17	5.3	59	19.8	12	7.0	88	11.1
		Some college	3	0.9	2	0.7	4	2.3	9	1.1
		Completed college	8	2.5	13	4.4	5	2.9	26	3.3
	Age group	< 20 years	17	5.3	12	4.0	3	1.8	32	4.1
		20–30 years	148	46.1	78	26.2	83	48.5	309	39.1
		31–40 years	59	18.4	42	14.1	43	25.1	144	18.2
		41–50 years	45	14.0	52	17.4	25	14.6	122	15.4
		51–60 years	27	8.4	45	15.1	15	8.8	87	11.0
		> 60 years	24	7.5	69	23.2	2	1.2	95	12.0
Provider survey		Total participants	n = 6		n = 4		n = 7		n = 17	
	Gender	Female	2	33.3	3	75.0	6	85.7	11	64.7
		Male	4	66.7	1	25.0	1	14.3	6	35.3
	Education	Some Secondary School	1	16.7	1	25.0	0	0.0	2	11.8
		Completed Secondary School	0	0.0	0	0.0	1	14.3	1	5.9
		Completed college	5	83.3	3	75.0	6	85.7	14	82.4
	Age group	20–30 years	4	66.7	2	50.0	3	42.9	9	52.9
		31–40 years	0	0.0	1	25.0	3	42.9	4	23.5
		41–50 years	1	16.7	1	25.0	0	0.0	2	11.8
		51–60 years	1	16.7	0	0.0	1	14.3	2	11.8
Observations		Total participants	n = 61		n = 23		n = 94		n = 178	
	Gender	Female	27	44.3	8	34.8	32	34.0	67	37.6
		Male	34	55.7	15	65.2	62	66.0	111	62.4
Drug bag interviews		Total households	n = 10		n = 10		n = 10		n = 30	
	Gender	Female	6	60.0	8	80.0	4	40.0	18	60.0
		Male	4	40.0	2	20.0	6	60.0	12	40.0
	Education	None	1	10.0	0	0.0	1	10.0	2	6.7
		Some Primary School	0	0.0	4	40.0	2	20.0	6	20.0
		Completed Primary School	9	90.0	6	60.0	7	70.0	22	73.3
	Age group	20–30 years	0	0.0	0	0.0	3	30.0	3	10.0
		31–40 years	0	0.0	0	0.0	6	60.0	6	20.0
		41–50 years	4	40.0	3	30.0	0	0.0	7	23.3
		51–60 years	2	20.0	2	20.0	1	10.0	5	16.7
		> 60 years	4	40.0	5	50.0	0	0.0	9	30.0

Note that for the drug bag, participant demographics presented here only represent one household member who was not interviewed alone but often supported by the spouse and/or children or other relatives present. Furthermore, during observations, participants' age or education was unknown but a gender was assigned by observers. All numbers are given as counts and as the percentage (%) of the indicated total participants (n)

### Characteristics of self-treatment

All human and veterinary health providers we surveyed in the study area dispensed various antibiotics. Although retail providers who are allowed to sell antibiotics can only do so upon presentation of a prescription, seven of the eight (87.5%) human retail providers we interviewed stated that the antibiotics they sell are all available over the counter. Even when they were aware of the requirement for a prescription, they never refused to sell antibiotics to clients who did not have one. During the exit survey, 40.7% (n = 337) of participants who bought an antibiotic in a human retail outlet or an open market reported that they had not seen a doctor before and 41.3% indicated that they did not have a prescription. Furthermore, during observations, 93.5% (n = 46) of the clients visiting human retail outlets who bought antibiotics did so without presenting a prescription. Drug bag interviews confirmed this practice as respondents mentioned repeatedly that they often go to pharmacies to buy the antibiotics they need without visiting a doctor first to obtain a prescription. Specifically, in 66.7% (n = 30) of the households we visited, interviewees said that they obtain a prescription only sometimes, while 16.7% (n = 30) said they never do.

Similar to the human retail outlets, all agrovet stores stocked and sold antibiotics to clients over the counter. During the exit surveys, all clients (n = 20) who bought veterinary antibiotics in a retail shop or at the market told us that they had received a recommendation from a veterinary officer. However, it was not clear if these recommendations pertained to the case at hand or had been given to participants at some point in the past. Meanwhile, in 66.7% (n = 30) of the households visited for the drug bag activity, respondents stated that they usually do not consult a veterinarian before buying antibiotics.

**Fig. 1:**
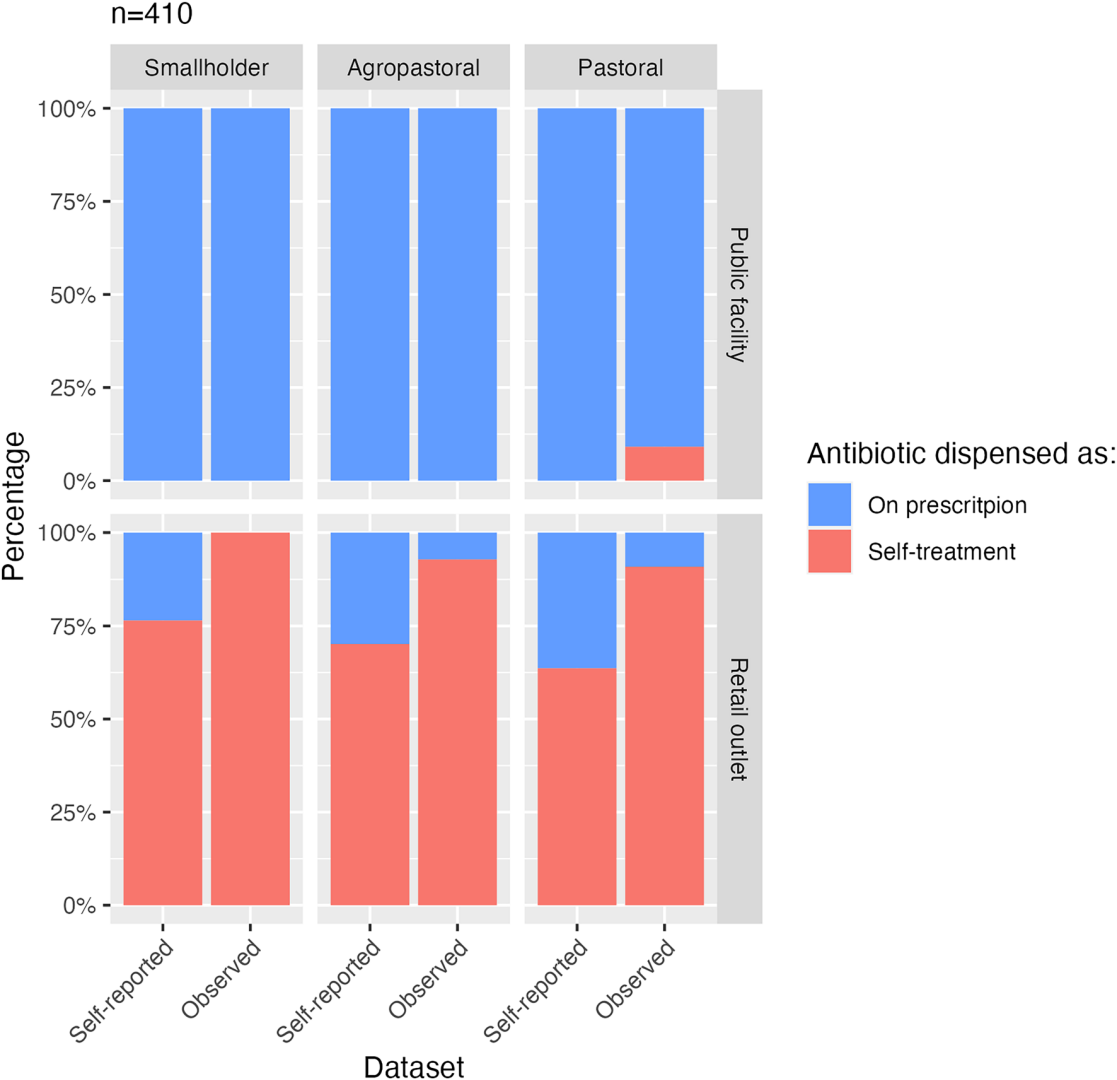
Rate of self-treatment with human antibiotics. Number and percentage of participants in the exit survey (self-reported) and observations (observed) who visited either public facilities or human health retail outlets and were dispensed antibiotics, either with a prescription or without, i.e. as self-treatment. Data is derived from: 1) Exit survey in both public and retail human health facilities (n = 337, 33.5% of smallholders, 49.3% of agropastoralists, 17.2% of pastoralists), representing the self-reported data; and 2) observations in both public and retail human health facilities (n = 73, 16.4% of smallholders, 38.6% of agropastoralists, 45.2% of pastoralists), representing the observed data

Self-administration of human antibiotics without a prescription was common across all districts with an overall self-reported rate of 41.0% (n = 337) of all antibiotics dispensed as self-treatment (28.3% (n = 166) of agropastoralists, 69.0% (n = 113) of smallholders and 24.1% (n = 58) of pastoralists). While all antibiotics in the public sector required a prescription for dispensing, a significant proportion (72.8%, n = 191) were dispensed without a prescription in retail outlets. The rate of dispensing without a prescription was similar among retail outlets in all districts, 76.5% (n = 102) in the smallholder district, 70.2% (n = 67) in the agropastoral district and 63.6% (n = 22) in the pastoral district. Observations hinted at a higher rate of self-treatment: 60.3% (n = 73) of all antibiotics were dispensed without a prescription (including 46.4% [n = 28] of agropastoralists, 83.3% [n = 12] of smallholders and 63.6% [n = 33] of pastoralists). Rates based on observations of clients purchasing antibiotics in retail shops without a prescription were even higher, with an overall rate of 93.5% (n = 46). This rate varied among districts. Specifically, 92.9% (n = 14) of clients were observed engaging in this practice in the agropastoral district, 100% (n = 10) in the smallholder district and 90.9% (n = 22) in the pastoral district (Fig. 1).

Farmers also engaged in self-treatment of animals with antibiotics. Although livestock field officers (LFOs) do not give out prescriptions per se, regulations suggest that farmers should wait for administration by the LFO. These regulations are seldom used in practice. Thus, for animal treatment, seven smallholders, six agropastoralists and seven pastoralists engaged in this practice. Our observations confirmed this practice: 13 pastoralists purchased antibiotics from retail shops and open markets for animal treatment. The findings suggest a higher occurrence of self-treatment of animals with antibiotics in pastoral regions, which aligns with the responses of drug bag respondents. All ten pastoral households where drug bag interviews were conducted mentioned this practice, as exemplified by the following quote:

Interviewer: “What first action do you take when your animals show symptoms of sickness?”

Respondent: “I start to inject it, give it medicine. […] I have them already stored here in the house.”

Interviewer: “You do not look for a veterinarian?”

Respondent: “Not usually.”

-Pastoral household

Antibiotics bought over the counter by study participants are summarised in Table 3. Across all datasets, the antibiotics most often bought without a prescription were amoxicillin (14.1%, n = 440), sulphamethoxazole/trimethoprim (12.3%, n = 440) and metronidazole (7.0%, n = 440) in humans. Antibiotics most commonly purchased for animals were oxytetracycline (61.9%, n = 63) and penicillin/streptomycin (30.2%, n = 63) (Table 3). Antibiotics for human use bought without prescription included Access and Watch antibiotics as well as formulations not recommended for use. Veterinary antibiotics fell within classes D, C and B. No Reserve (humans) nor class A (animals) antibiotics were identified.

**Table 3:** Antibiotics available in retail outlets and the number and percentage of respondents who bought them without presenting a prescription

Antibiotics available in retail outlets dispensing antibiotics for human use	AWaRe classification	Bought without prescription in:			
		Self-reported		Observed	
		Exit survey	Drug bag interview	Observations	Total
		n = 337	n = 30	n = 73	n = 440
Amoxicillin	ACCESS	33 (9.8%)	17 (56.7%)	12 (16.4%)	62 (14.1%)
Amoxicillin/Clavulanic acid	ACCESS	0 (0.0%)	0 (0.0%)	0 (0.0%)	0 (0.0%)
Ampicillin	ACCESS	5 (1.5%)	0 (0.0%)	2 (2.7%)	7 (1.6%)
Cefalexin	ACCESS	1 (0.3%)	0 (0.0%)	1 (1.4%)	2 (0.5%)
Chloramphenicol	ACCESS	3 (0.9%)	1 (3.3%)	0 (0.0%)	4 (0.9%)
Doxycycline	ACCESS	12 (3.6%)	1 (3.3%)	3 (4.1%)	16 (3.6%)
Gentamicin	ACCESS	0 (0.0%)	3 (10.0%)	0 (0.0%)	3 (0.7%)
Metronidazole	ACCESS	17 (5.0%)	3 (10.0%)	11 (15.1%)	31 (7.0%)
Nitrofurantoin	ACCESS	0 (0.0%)	0 (0.0%)	0 (0.0%)	0 (0.0%)
Penicillin V	ACCESS	9 (2.7%)	4 (13.3%)	3 (4.1%)	16 (3.6%)
Penicillin G	ACCESS	0 (0.0%)	0 (0.0%)	0 (0.0%)	0 (0.0%)
Sulphamethoxazole/Trimethoprim	ACCESS	37 (11.0%)	8 (26.7%)	9 (12.3%)	54 (12.3%)
Tetracycline	ACCESS	4 (1.2%)	5 (16.7%)	0 (0.0%)	9 (2.0%)
Oxytetracycline	ACCESS	0 (0.0%)	0 (0.0%)	0 (0.0%)	0 (0.0%)
Azithromycin	WATCH	1 (0.3%)	0 (0.0%)	0 (0.0%)	1 (0.2%)
Ceftriaxone	WATCH	2 (0.6%)	0 (0.0%)	1 (1.4%)	3 (0.7%)
Ciprofloxacin	WATCH	3 (0.9%)	1 (3.3%)	2 (2.7%)	6 (1.4%)
Erythromycin	WATCH	6 (1.8%)	0 (0.0%)	2 (2.7%)	8 (1.8%)
Minocycline	WATCH	0 (0.0%)	0 (0.0%)	1 (1.4%)	1 (0.2%)
Neomycin	WATCH	0 (0.0%)	0 (0.0%)	0 (0.0%)	0 (0.0%)
Ampicillin/Cloxacillin	NOT REC	15 (4.5%)	3 (10.0%)	1 (1.4%)	19 (4.3%)

Antibiotics available in retail outlets dispensing antibiotics for veterinary use	ABCD classification	Bought in:			
		Self-reported		Observed	
		Exit survey	Drug bag interview	observations	Total
		n = 20	n = 30	n = 13	n = 63
Amoxicillin	D	1 (5.0%)	0 (0.0%)	0 (0.0%)	1 (1.6%)
Oxytetracycline	D	12 (60.0%)	18 (60.0%)	9 (69.2%)	39 (61.9%)
Sulfadimidine	D	2 (10.0%)	1 (3.3%)	0 (0.0%)	3 (4.8%)
Sulphamethoxazole/trimethoprim	D	0 (0.0%)	0 (0.0%)	1 (7.7%)	1 (1.6%)
Chlortetracycline	D	0 (0.0%)	0 (0.0%)	1 (7.7%)	1 (1.6%)
Penicillin/streptomycin	C	3 (15.0%)	14 (46.7%)	2 (15.4%)	19 (30.2%)
Tylosin	C	2 (10.0%)	0 (0.0%)	0 (0.0%)	2 (3.2%)
Enrofloxacin	B	1 (5.0%)	3 (10.0%)	2 (15.4%)	6 (9.5%)
Levofloxacin	B	1 (5.0%)	0 (0.0%)	0 (0.0%)	1 (1.6%)

These data incorporate the different datasets assembled for this study, including self-reported and observed data. The number of respondents who reported or were observed to access a specific antibiotic without prescription is shown in the first row (n). The Access, Watch, Reserve (AWaRe) classification of antibiotics for human consumption [47] and the ABCD categories, namely Avoid, Restrict, Caution and Prudence, for veterinary antibiotics [48], are used to show the importance of each antibiotic class for human health and their potential impact on public health. ACCESS and class D antibiotics are first-line treatments, while WATCH or class C or B antibiotics should be used more selectively. Not recommended or class A antibiotics should not be used at all

### Healthcare associated drivers of self-medication

A number of drivers of self-treatment linked to inadequacies in access to healthcare were identified during interactions with respondents. Findings are presented based on the well-documented themes developed as part of a framework for examining access to quality healthcare in low-and-middle income countries [5, 39].

#### Availability

Despite the presence of a public health facility in each village, a significant majority of households (93.3%, n = 30) reported not consistently obtaining antibiotics from there and instead opting to seek them at pharmacies at times. In 73.3% of drug bag interviews (n = 30), participants explicitly said that one of the reasons for buying antibiotics at the pharmacy were drug stockouts in the public health facilities. While stockouts also happened in retail outlets, the greater availability of drug or agrovet shops compared to health care facilities meant that the needed drug could be found somewhere else. Amongst providers, 33.3% (n = 3) of public (human) providers, 87.5% (n = 8) of retail providers dispensing human drugs and all veterinary retail providers (n = 6) reported experiencing stockouts of at least one of their three most popular antibiotics. This is most likely an underestimate, especially for public facilities, as, in 58.9% (n = 192) of exit interviews, participants stated that they could not obtain all the medicines they needed from the public facility they had just visited. During observations at a dispensary, there were five patients in one day who were sent to a pharmacy to buy painkillers or erythromycin as these were out of stock at the public dispensary. This situation was also described by our study participants during interviews:

Respondent: “At the hospital you are often told there is no medicine. You get there and you are told we don’t have this medicine, … but in the pharmacy they are available.”

-Smallholder household

Respondent: “There is a challenge [with medicine availability], even now. But it is better now, not like in the past when we used to depend on the medicines from the dispensary where you find they are out of stock. Nowadays there are drug shops, so it has been a bit better. Because when you miss [the medicine] at the hospital you can go to the drug shops. The shops are very many.”

-Pastoral household

In the case of animal health issues, farmers stated that when they called the LFO—the public extension officer—to their homes, they usually came with the needed antibiotics. However, they also reported that many times the LFO is not available. They are, therefore, forced to manage the diseases of their livestock themselves with drugs purchased at agrovet stores and administered by themselves, as this respondent told us:

Interviewer: “Do you usually go to the veterinary officer first [before buying oxytetracycline injection], so that he instructs you on how to use it or you do it yourself?”

R: “No. Where will you get him here madam? Where will you get him? You will just delay things. You will find your cow dead. We have already become veterinary officers ourselves [laughter]. We have syringes. Almost wherever there is a family with livestock, you will find that they have a syringe.”

-Agropastoral household

In some instances, the extension officer advised farmers over the phone and recommended drugs to buy, as explained in this conversation:

Interviewer: “Is it possible that the [extension officer] is late, or is away sometimes?”

Respondent: “No, he leaves his phone numbers, so you can call him and talk to him. He will direct you [about what to do].”

-Smallholder household

Participants often referred to agrovet owners or other local people perceived as professionals in livestock or agricultural matters as “livestock doctors”, although it was not always clear what kind of certification or skills such local professionals have (see Virhia et al. [15] for a more detailed description of various animal health providers). Such animal health specialists, either available through the government or operating privately, were relied upon mostly by smallholder farmers, as illustrated in this dialogue:

Interviewer: “When you need a veterinarian when your livestock is sick, is it a service that you get easily?”

Respondent:” Sometimes it is not so easy. But we often get [service] because there are other veterinarians who are not from the government. There are people who have studied veterinary medicine as well. […]”

Interviewer : “So those also help?”

Respondent : “Those are the ones that help us a lot.”

-Smallholder household

Generally, antibiotics were perceived to be readily available, especially in retail outlets. Few drug bag respondents had encountered difficulties in sourcing antibiotics for use in humans (13.3%, n = 30) or animals (10.0%, n = 30) in the past. In the exit survey, 93.9% (n = 478) and 98.6% (n = 71) of participants visiting a drug shop or an agrovet store, respectively, reported to have bought all medicines that they needed.

#### Accessibility

**Fig. 2:**
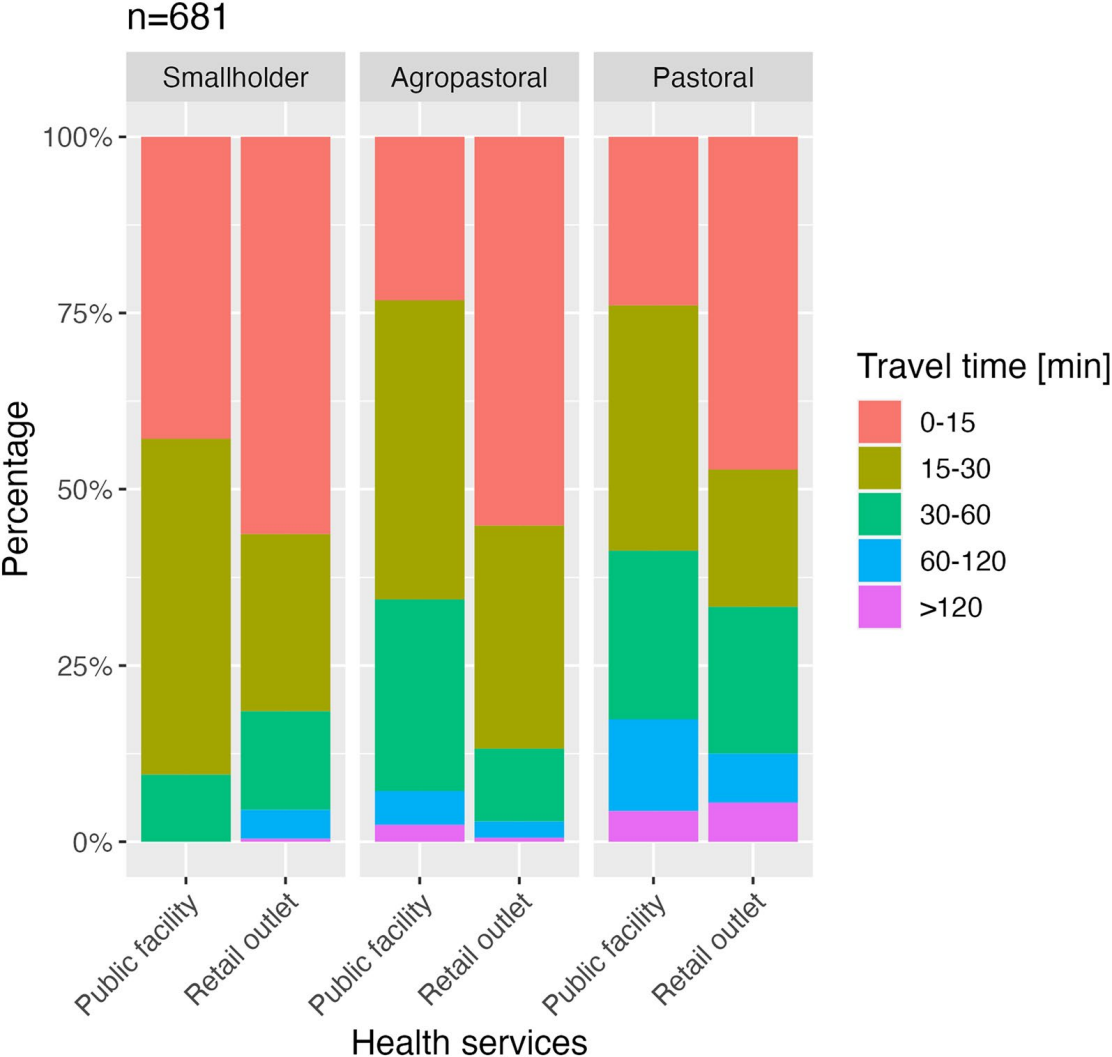
Travel time to human health services. Stacked bar chart showing the travel time to different human health services reported by study participants presented as proportions of respondents by travel time. Public facilities refer to the public health facilities including dispensaries and health centres, whereas retail outlets are pharmacies and other drug dispensing shops. Data are derived from the exit survey with a total of 681 participants, including 264 (43.9%) smallholders, 299 (38.8%) agropastoralists and 118 (17.3%) pastoralists

Study participants reported greater accessibility of retail outlets compared to public health facilities. The mean time to reach a health facility reported in the exit survey was 41.2 min versus 26.4 min for a retail outlet. In smallholder areas, the mean time to reach a health facility was 24.2 min which is comparable to the mean time to reach a retail outlet (24.5 min). In the other districts, the way to a public health facility was longer than the way to a retail outlet (34.0 min vs 23.3 min in agropastoral and 52.3 min vs 40.4 min in pastoral regions). Travel times to different health facilities in all districts are illustrated in Fig. 2.

Most respondents (67.0%, n = 708) travelled by foot to reach healthcare facilities. Other means of transport like the motorcycle, bus or a car bear a cost which is not always affordable as shown in the quote below. In that case, the respondent then resorted to counselling over the phone.

Interviewer: “If you are not seriously sick, you can just call the doctor and he gives you advice?”

Respondent: “Yes.”

Interviewer: “That you go and buy certain medicines?

Respondent: “Yes, but mostly he doesn’t like that, he wants to see me in person. But I tell him, please I don’t have [resources for] the bus fare. Because that is just up to you [to pay], you will need to find [the money] somewhere. It is up to you.”

-Smallholder household

For veterinary issues, the LFO usually visits people’s homes, although his accessibility was perceived very differently across study sites. For instance, participants from smallholder areas usually felt that the LFO or local livestock experts were easily accessible and only a phone call away, as this respondent from a smallholder area explained:

Interviewer: “And the livestock officer, every time you call him he is around? Or how is his availability?”

Respondent: “We have the government livestock doctors and private livestock doctors that we see passing on the streets. They have these medicines [antibiotics]. He says I treat livestock, maybe he worked in the past and retired, returned home, and now he is continuing to help the citizens here. Therefore, we trust them.”

Interviewer: “So you don’t have problems accessing the cattle doctors? They are present?”

Respondent: “Yes, if you want the government one, you request him at the division, and he comes.”

Interviewer: “And if you want the private one is he available too?”

Respondent: “Yes.”

-Smallholder household

By contrast, farmers in pastoral areas preferentially chose to self-treat with medicines bought at an agrovet store as a veterinarian would not be able to visit them promptly. In these areas, none of the respondents mentioned LFOs or other local livestock experts as the first port of call when their animals are sick. In comparison, agrovet stores, which are typically present in every village, were perceived as a very accessible and reliable source of medicines in all villages. One pastoralist explained to us the first steps he takes when he notices that his animals are sick:

Respondent: “When I first see the symptoms, I quickly go to the agrovet and find medicines to treat them.”

Interviewer: “Is there anything else you do, when you discover that your animals are sick?”

Respondent: “I do nothing else, when I see [they are sick], I just get prepared with the drugs.”

-Pastoral household

#### Affordability

Public health services were perceived as being costly, for both humans and animals. For human health matters, while insurance or waivers can be deployed at public health facilities, people are often sent to shops with prescriptions from the doctor due to frequent drug stockouts. The double cost of paying for insurance and the medicines at the retail outlet can amount to a prohibitive sum, as, for example, this respondent told us:

Respondent: “If you don’t have insurance you better go and get advice from the pharmacy, because if you go there [to the dispensary] money is wasted. You are sent to the pharmacy again and you spend more money. So, you see that it is better to start at the pharmacy. You just explain your problem, buy the drugs, that is all. Rather than go to the hospital and use more money, and then go looking for more money [to buy the drugs at the pharmacy].”

-Pastoral household

Therefore, 16.7% (n = 30) of our participants mentioned paying for health insurance, but many more complained about its unfeasibility.

If the facility is far away, which is more often the case for public facilities, as presented above, then the cost of transport needs to be considered as well. To minimise costs, many people bypass the public health system by directly buying what they need (or want) from retail outlets, especially if they are familiar with the symptoms they are experiencing. This is a cheaper alternative compared to visiting a public health facility, as this respondent explains:

Respondent: “We just go to buy drugs from the pharmacy. If you go to the hospital, then only with money! We are told, with money!”

Interviewer: “But in the pharmacy they also ask for money, no?”

Respondent: “In the pharmacy it is less money.”

-Agropastoral household

Similarly, paying the LFO to visit your home often means reimbursing his travel costs, i.e. petrol or taxi services, in addition to paying for the examination and/or treatment. Some of our participants proposed fixed rotational visits to households by the LFO. This would help them plan for expenses and take action against disease early, for example by implementing preventive measures, as advised by a professional. Another solution to save money is to prepare or buy alternative medicines like herbal drugs, for example. Some participants explained that they would first try to cure the condition themselves by applying herbs before attending a medical outlet. Others always treat specific diseases with herbal remedies. In veterinary care, chickens were not considered sufficiently valuable to require expensive injectables from the agrovet store and are therefore given aloe vera or other plant- or food-based remedies instead. Herbal remedies were mentioned in all study areas, but more often in smallholder households, as illustrated by the quote below:

Respondent: “Injection [insulin for diabetics] is very expensive. And our economic situation is difficult. Buying that small bottle and inject it every day, it is very expensive. So that is the reason why people shift to local herbs, because of the cost. The cost of the medicine is very high.”

-Smallholder household

Costs were described as an impediment also by providers. For example, 81.8% (n = 11) and 33.3% (n = 6) of attendants at human and veterinary drug outlets, respectively, mentioned that not all clients visiting their shops could always afford a full dose of antibiotics. The exit survey results confirmed this, with 20.7% (n = 319) of participants reporting buying only a partial dose of antibiotic in human drug shops. This practice was also mentioned in the drug bag interviews, as shown below:

Interviewer: “And how many times do you take amoxicillin, maybe three, four or five days?”

Respondent: “They can give you a dose of five days. But if you go to the shop they sell it to you in cash, so you can decide not to use a full dose, depending on the money you have.”

-Pastoral household

Others said they would use left-over medicines they still had at home from earlier courses, like this participant, for example:

Interviewer: “So, you go on to use the rest [of the medicine] you stored at home?”

Respondent: “Yes, you can use it so long as you are aware of how long it has been stored for. And you can use it because of financial problems. Sometimes you find that you have no money to go and buy new medicine. You find it is better to take the one you have preserved from last time.”

-Agropastoral household

For veterinary drugs, to save money, farmers sometimes pooled money together to buy a bottle of antibiotic or chose smaller packages or tried to haggle over drug prices. Alternatively, smallholder households, in particular, bought human antibiotics instead and gave these to chickens through water or feed. This reduces costs, as these antibiotics can be bought in single capsules or tablets rather than in a big bottle. Another strategy employed was to buy veterinary antibiotics when money was available and to store them at home for usage in times of scarcity, as this participant explained:

Respondent: “If you see [the animal] is sick, and the drug is there [at home], you just treat it. Because if you go to tell the livestock officer that my cow is sick, he wants payment if he comes to treat it. But you have bought the drug, so it is available and you know how to treat. You just inject it and your cow or goat gets better. The drug is there because you bought it earlier. If you know what fever this goat has, you just treat it. If you go to call the officer, your pocket must be full.”

-Agropastoral household

#### Adequacy

Long waiting times often caused by staff shortages are a problem mostly experienced by patients in public health facilities. While all participants in the exit surveys only spent up to 15 min in a pharmacy or a shop, 41.3% (n = 191) spent more than two hours at a public health facility. This can discourage attendance as this respondent explains:

Respondent: “You will get to the hospital and waste time, you will waste time! Maybe you arrived at 8:00am and waited up to 10:00am, the doctor probably is the one who is coming in late. Now at 10:00am he is here, but until they get to you, at the end you are told we don’t have the medicines, go to buy them there [at the pharmacy]. You are wasting your time, and see, when I feel bad, I rather run to the pharmacy, [the medicines] are available there and you are served immediately.”

-Smallholder household

During observations, the mean time between entering and leaving a facility for clients who were dispensed a medicine was 3.1 min in human and 4.4 min in veterinary retail outlets, respectively. In human public facilities, the time spent was slightly higher (mean of 9.6 min). However, this did not include the time spent with the doctor.

In addition, as we observed during our field visits, opening hours were very restricted at public facilities which usually closed at around 3:30 pm. Pharmacies and shops, in contrast, were open all day and often catered for clients who needed to attend early in the morning or late in the evening to be able to carry out farming work during the day.

#### Acceptability

Medical doctors were seen as the most acceptable source of health provision. Medical drugs, and specifically antibiotics, were known and trusted to work by all households, for use both in humans and animals. However, diagnostic tests were mentioned by our participants to be necessary to decide on treatment for people, but not for animals, as illustrated in this quote from a pastoral household:

Respondent: “All these medicines, I think they are important, but you need to have a blood test. If a person gets a blood test, that is when the medicine will be used for the right disease in humans.”

-Pastoral household

There was widespread awareness that the correct course of action is to visit a doctor or call the LFO before buying medicines. Not doing that could have bad consequences as this respondent explains:

Respondent: “When you just go to the shop and buy the medicine you are not sure what it treats. But at the hospital, there the doctor tests and knows you have this problem. He prescribes a medicine that ends that problem. […] It might be tuberculosis and you are taking amoxicillin, you need to go to the hospital, get tested to know this is tuberculosis. The doctor asks you, when did you start [to take the medicine] and he even takes the medical history: When did you start coughing, when did the chest problem start? You explain to him what medications you have already taken. You explain - it is like you are interviewing me now - and at the end he knows what disease it is. He tests you and he tells you the problem is this, use these drugs. He may prescribe you amoxicillin and paracetamol or others, to use in the time he has given you and if you feel the body is feeling well, that’s it.”

-Smallholder household

LFOs and veterinarians were the most trusted sources of veterinary health provision, especially in smallholder areas. Here, people often fully relied on either the LFO or local expert to diagnose and treat the conditions their animals suffered from, like this respondent, for example, illustrated:

Respondent: “Often, when you call the LFO it is good, because you will get advice, rather than deciding for yourself. When you feel in your body, this is fever, and go to buy medicine at the drug shop, that’s one thing, but for the cattle you don’t know what problem it has and so you have to call [the LFO].”

-Smallholder household

In agropastoral settings, participants with no experience of treating animals behaved very similarly and often bought and administered medicines from agrovets only after communication with a trusted local livestock expert or the LFO. In contrast, pastoralists and agropastoralists with big cattle herds expressed much lower reliance on public veterinary services. Trust in the LFO was still present but with some reservations. Farmers valued their own experiences more than in other areas and often did not feel a need for consulting a local livestock expert or the LFO, as illustrated in the quote below. In three cases, respondents did not even mention the LFO during the whole drug bag interview when discussing antibiotics.

Interviewer: “And what do you do in the beginning when you notice that an animal is sick?”

Respondent: “First of all you can decide, you might get advice from the LFO, but we have also learned what to do when an animal has a certain disease, so we know which medicines can cure it.”

Interviewer: “So, you keep drugs [antibiotics] at home [to treat yourself]?”

Respondent: “Yes, we know everything: Which disease it is, and which treatment is needed.”

-Pastoral household

Another acceptable treatment option were herbs instead of medical drugs. Some respondents, like the one quoted below, told us that they work well to treat certain diseases. Generally, it was not common to take medical and herbal drugs at the same time. Some respondents preferred herbal remedies, especially for treatment of chronic disease like high blood pressure or diabetes, for which herbs sometimes were seen to treat better than medical drugs, as the respondent quoted below stated:

Interviewer: “What drugs do people use in your household to treat diseases [that are present in your household]?”

Respondent: “First, we use traditional medicine. If you then go to the hospital with a fever, there are other medicines [medical drugs] you are given.”

Interviewer: “So, which one do you like more the hospital or traditional medicine?”

Respondent: “The diseases we use them for are different. For example, there are those that we only treat with traditional medicine, like stomach ache. […] So, we often use traditional medicine, and it helps us to treat the disease.”

-Pastoral household

### The role of informal health seeking networks

Even though study participants often self-treated without advice from a medical professional, they always made an effort to obtain information about which treatment to choose and how to use it in ways accessible to them. When the formal option—consulting the doctor or LFO—was not available or accessible, they made use of informal networks of trusted people, for example treatment vendors, family members or neighbours, to discuss health choices. Furthermore, their own knowledge, prior experiences and old prescriptions often guided them when assessing the value and veracity of advice from such sources, and when ultimately deciding on a health seeking or treatment pathway.

Retail vendors were accessed very often for assistance on treatment choice and usage, but their advice was also often seen as not ideal and biased. People were wary of hidden business incentives and sometimes perceived vendors as trying to sell them too many or very expensive products to make more money or dispensing expired drugs in order to sell old stocks, as this respondent described:

Interviewer: “Do you trust the medicine vendors in the agrovets?”

Respondent: “Sure, I can trust them, but I also know that those are businessmen. So, I might decide to visit the agrovet because my cow is sick. I can buy a medicine […] without considering the vendor’s opinion, as he/she might even tell me to buy the whole agrovet. So, I only consider my problem at that moment. They are not very much trustworthy.”

-Pastoral household

Local veterinary experts or drug vendors were called upon for advice on diagnosis, treatment choice and drug use in animals, even though respondents were aware that some of them might not have the necessary background or knowledge to advise them well, as shown by the two quotes below. Similar reservations were expressed regarding advice from friends or neighbours, who were often not seen as educated or knowledgeable enough to advice on treatment modalities.

Respondent: “The pharmacist, when you go to the pharmacy and if he has gone to school, then that’s ok. But if he is not educated, he will advise you as he will advise you. And that is why sometimes we use the medicines and they harm us.“

Interviewer: “But you still take advice from the seller sometimes?”

Respondent: “He can advise you ‘use this, it will help you’, but you can’t know if he is hundred percent right, because maybe he is not educated. He just talks because he is in his workplace, that’s how it is.”

-Smallholder household

Respondent: “There are the private ones [local veterinary experts]. But most of the time we don’t depend on the private one, because they might kill your cow. Because they just work by guessing.”

Interviewer: “They don’t have the required knowledge?”

Respondent: “No, they are just given one seminar and then they come and treat the cows.”

-Smallholder household

In line with this sentiment, according to our data, only one out of six veterinary drug vendors had relevant educational background – a diploma in animal health -, whereas all (n = 11) human drug vendors had either certificates in nursing or community health or specialised training in drug dispensing.

Such informal networks, including vendors, local health experts, friends or neighbours, were often the best option many respondents had to help them decide on their health seeking choices. Neighbours and friends who had experienced similar symptoms/clinical signs in the past could give advice on treatment choice or they could pass on information a doctor, veterinarian or trusted informal source had given them. Respondents reported discussing symptoms/clinical signs and possible treatment with friends and family to decide when and where to seek healthcare. In pastoral households, it was especially common to self-diagnose people and animals and decide on the type of treatment with the help of neighbours and friends (50.0% [n = 10] of respondents in pastoral versus 33.3% [n = 10] and none in agropastoral and smallholder areas, respectively). It was also common practice to borrow some of the needed antibiotics, especially for the treatment of animals. However, this occurred also for the treatment of human conditions, as expressed by this pastoral respondent:

Interviewer: “Who gives you good instructions for using [tetracycline eye cream for human use]?”

Respondent: “[The information] must come from the place where you took the medicine from, if it is in the hospital, they do give instructions. If it is from the shop, they give you instructions. Even if you get it from the neighbour, they direct you.”

-Pastoral household

[Talking about a time when an animal was sick:]

Respondent: I asked a friend if he had medicines and he went to give me this [oxytetracycline 10%]. When I was taking it [home], there was somebody else who advised me to go for [oxytetracycline 33%]. Then I got that other one.

I: So, you got advice from a neighbour?

Respondent: From a fellow livestock keeper.

-Agropastoral household

Self-acquired knowledge was also utilised to make health seeking decisions. For example, in cases of reoccurring diseases, participants said they had become used to the treatment they had received repeatedly for the same condition. Some participants had used a specific antibiotic so often that they stated to “know” the drug well, or even that they had become “like an expert of a certain drug” and its usage, like the participant quoted below.

Respondent: “It has become something that I know generally, when you see that the eyes or ears trouble you, you just look for [gentamicin drops], because you will have been given that in the past [for the same symptoms]. You even find the old box and go with it [to the pharmacy] to buy it again. […] You just become like an expert of a certain drug, like when we take these [drops] we have to do it like this.”

-Agropastoral household

Therefore, instead of visiting a medical professional, they would go directly to a retail outlet to request the same medicine which they would use as directed in older prescriptions or based on recommendations by medical professionals.

Interviewer: “Who directs you how to take this medicine [amoxicillin]?”

Respondent: “Because you have already gone to the hospital the other day [in the past], and you were prescribed the same and used it, therefore, when you go to the pharmacy…”

Interviewer: “You just follow the previous prescriptions you were given by the doctor?”

Respondent: “Yes. Even when the pharmacy attendant prescribed it wrongly, I will be able to know because the doctor has told me otherwise.”

Interviewer: “You will be able to know the doctor told me this, so you follow the previous prescriptions?”

Respondent: “Yes.”

-Smallholder household

**Fig. 3:**
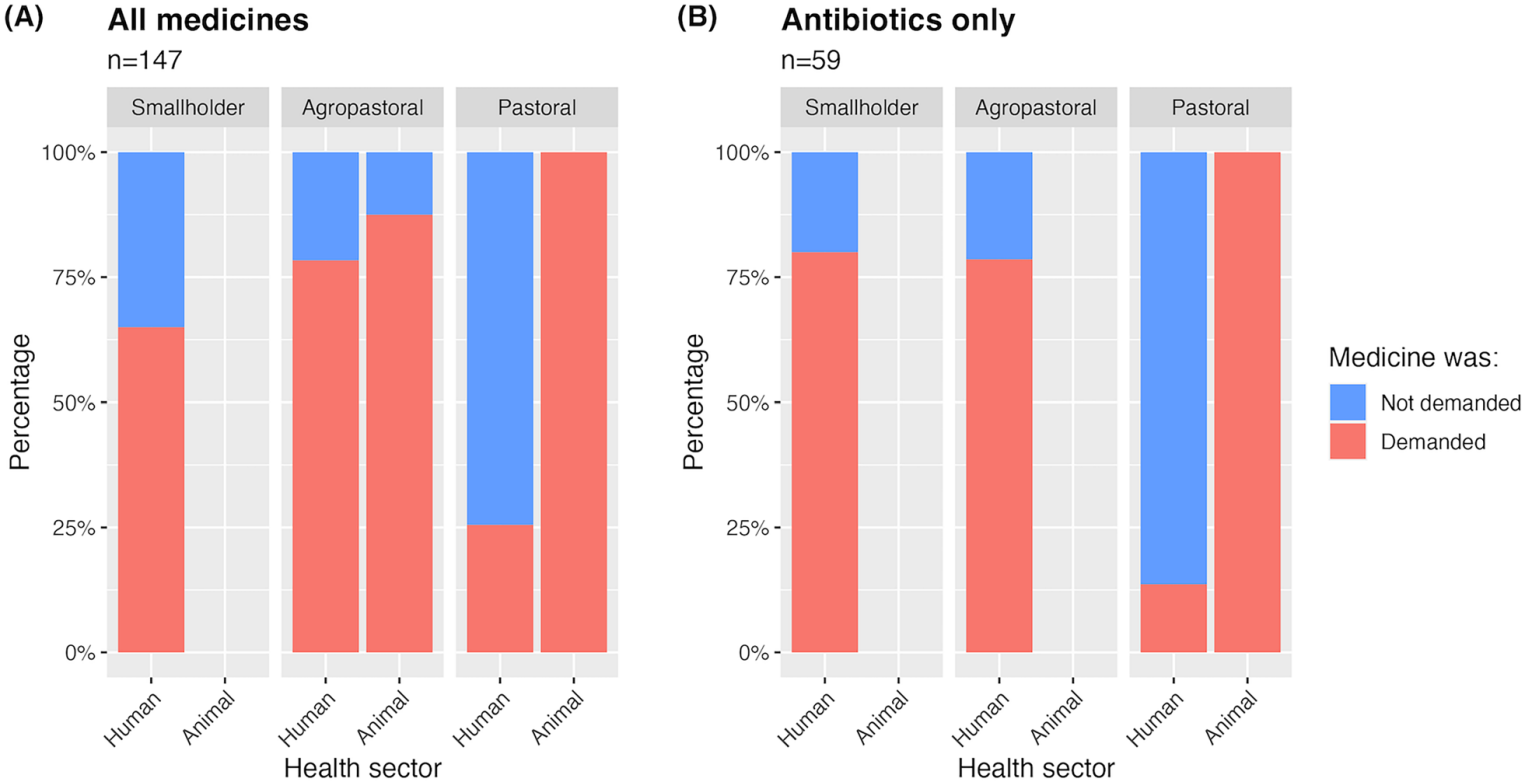
Rate of clients asking for specific non-prescribed treatments. Stacked bar charts showing the percentage of participants who asked for a specific treatment without presenting a prescription nor asking for help on drug choice by the vendor during observations. **A** shows demand for any medicines (n = 104 for human and n = 43 for animal treatments). **B** is a subset of **A** and shows demand for antibiotics specifically (n = 22 for human and n = 13 for animal treatments). Observations (n = 147) included 47 pastoral respondents at human and 35 at animal health outlets, 37 agropastoral respondents at human and eight at animal health outlets, and 20 smallholder respondents at human and no smallholder respondents at animal health outlets. In two days of observations, no client visited the animal health outlet in the smallholder village to buy any veterinary medicines. Therefore, no observations could be made. In agropastoral regions, observations were made but no client bought an antibiotic

Our observations illustrate this point further. In human retail outlets, we observed that 51.9% of clients (n = 134), including 12 (25.5%) pastoralists, 29 (78.4%) agropastoralists and 13 (65.0%) smallholders asked for a specific medicine without presenting a prescription or asking for advice from the vendor. These included 22 (16.4%) clients—3 (13.6%) pastoralists, 11 (78.6%) agropastoralists and 8 (80.0%) smallholders -, who asked for antibiotics specifically (Fig. 3). In veterinary outlets, this practice was even more pronounced: all pastoralists (n = 35) and 87.5% (n = 8) of agropastoralists asked for specific drugs. For example, all pastoralists (n = 13) who were dispensed antibiotics had specifically requested medicines of this type (Fig. 3). Typically, clients were provided with the requested medicines, including antibiotics, without any additional consultation from the vendor to ensure that the treatment was suitable for the client's condition.

Regarding veterinary treatment, in the case of pastoralists, they relied extensively on their own long-term experience with diagnosing and treating their animals. They reported having knowledge about a wide range of livestock diseases, which became apparent by the clear and sophisticated descriptions of symptoms, causes and treatments needed, especially compared to households from other districts. Pastoralists reported identifying the needed drug and dosage themselves through trial and error or based on a combination of advice from sellers, leaflets and the LFO. One respondent said that every pastoralist in this region knows these medicines and they do not need advice from anyone on how or when to use them, as illustrated below:

Interviewer: “How do you know the amount to inject? Did the agrovet seller, the LFO or your neighbour tell you? Or do you have experience?”

Respondent: “As livestock keepers, we know this medicine. […] We have experience because we use these medicines all the time. We have discovered that for a sheep we inject perhaps 4 millilitres, but we consider the animal’s weight as well. […]”

Interviewer: “But have you ever received any advice from the LFO or the agrovet seller?”

Respondent: “No.”

Interviewer: “It is only a matter of experience?”

Respondent: “Yes.”

-Pastoral household

Interviewer: “How do you decide the dosage amount?”

Respondent: “I decide, I inject the animal and observe it for that day to see if it improves.”

Interviewer: “And how do you decide the amount?”

Respondent: “I have just gained that knowledge from keeping livestock a long time. I learned that if I inject a certain amount the animals will respond.”

-Pastoral household

Despite this perceived understanding, during the drug bag activity, we observed that antibiotics are erroneously classified as painkillers and antimalarials, and that veterinary antibiotics are confused with deworming drugs or vitamins in all districts.

## Discussion

Self-treatment is a widely practised approach to managing clinical signs/symptoms, particularly in regions with weaker healthcare systems and limited medicines control infrastructure [11]. However, this approach poses moral, ethical and public health challenges, particularly concerning the emergence of AMR to essential medicines. On the one hand, there is a duty to preserve the integrity of currently effective antibiotics. On the other hand, in the absence of adequate healthcare safety nets provided by governments, the ethics and morality of such efforts become questionable. In this study, we sought to examine the extent to which farmers and other community members medicate themselves and their animals in three representative livestock production systems of northern Tanzania—agropastoral, pastoral and smallholder—predominant across East Africa. To accomplish this, we used both quantitative and qualitative datasets. Our findings indicate a widespread practice of self-treatment, with an overall self-reported rate of 41% (69.0% of smallholders, 28.3% of agropastoralists, 24.1% of pastoralists) for human antibiotics. High rates of self-treatment were observed in human health retail shops: 76.5% among smallholders, 70.6% among agropastoralists and 63.6% among pastoralists. Self-treatment of animals was reported as well, with especially high frequency in pastoral areas. This is in line with previous literature: according to a review, self-treatment rates in humans in East Africa were at 47.1% [12]. Mdegala et al. [49] also suggest widespread antibiotic self-treatment by farmers in Tanzania. While most participants expressed a preference for receiving advice and a confirmatory diagnosis from a medical doctor or veterinarian when dealing with health issues within their households, they often faced obstacles in accessing official services in their daily lives. Another study conducted in northern Tanzania showed that, while most participants acknowledged regular self-treatment, they believed it was not as effective as seeking medical consultation [14].

Commonly reported issues around accessing health care included high costs, distance and time required to reach health facilities, unavailability of services or long waiting time. These have also been reported in other studies in the same region [14, 29, 31, 50]. When people visited public health facilities, they often did not receive complete services due to a lack of diagnostics, a shortage of professionals and medicine stockouts. At the same time, antibiotics were readily available in pharmacies and agrovets, constituting a viable alternative option for people to overcome these issues and treat themselves or their animals in due time, efficiently and at a reduced price. However, such self-treatment resulted in a lack of professional advice and counselling on drug choice and how to use the purchased medicines appropriately. Consequently, participants heavily depended on information retrieved from old prescriptions or friend networks to diagnose themselves or their animals and decide on a treatment course. Pastoralists in this study, specifically, often relied on their own experiences for diagnosing and treating their animals.

We show here that self-treatment occurs when people are not able to access adequate care and are therefore forced to resort to different ways of seeking healthcare. They search for advice from informal networks and gain experience around disease and treatment which helps them to navigate healthcare and treatment options available to them in an efficient, affordable and acceptable way. As such, self-treatment emerges from the interplay between structural, social and political factors, including existing health inequalities and inadequacies of the health system combined with historical marginalisation of rural communities. People use their informal networks to circumvent such barriers. However, concerns have been raised regarding the quality of health services through such networks, as treatment choice and information received through them is often incomplete or inaccurate [5, 18, 24, 30, 32, 51] endangering patient’s health and safety and possibly furthering AMR development or spread.

### The link between healthcare structural challenges and self-treatment

Because of such structural deficiencies, study participants were often unable to reach and access medical professionals to obtain a diagnosis and correct treatment advice for themselves or their animals. Such flaws in availability, accessibility, affordability, adequacy and acceptability of good quality healthcare are widespread in East Africa [5]. Green et al. [21] noted that such constraints are perceived throughout all socio-economic levels. Therefore, in order to reduce self-treatment, access to good quality public healthcare needs to be increased. This aligns with the sustainable development goal of ensuring healthy lives and promoting wellbeing for everyone, which includes attaining universal health coverage to ensure that everyone has access to the health services they need. In addition, people should be made aware of and incentivised to use such options when they are available. Similar programmes have been implemented with the goal to increase access and quality of malaria care in Tanzania. For example, Hetzel et al. [52] employed social marketing strategies as part of their ACCESS programme to boost the demand for quality services regarding malaria care. The programme also integrated multiple approaches to improve the quality of healthcare services and facilities, both public and private. These included training and supervising health facility personnel, implementing a quality management system in health facilities, and introducing licensed accredited drug dispensing outlets staffed with trained vendors authorised to sell first-line treatments.

Access barriers have been identified as the main reasons people do not utilise public health facilities [14, 21, 29, 32]. Removing these barriers is an essential first step to address issues around self-treatment and providing healthcare users with the agency to choose what they often already see as the best option for health seeking. In our study area, for human health issues, there is high awareness of the official way to access healthcare (starting at a public health facility), and of the need to obtain a prescription for medicines. This provides a strong basis for programmes to be developed. However, interventions to increase staff numbers and make public health facilities more accessible and affordable, and medicines as well as diagnostics more available at point of care are critical. In the case of veterinary services, the use of animal health specialists is much lower compared to self-treatment with antibiotics purchased at agrovet stores. Governmental veterinarians present are not enough to cover demand and private veterinarians do not reach rural areas, especially in pastoral regions [31]. Investment in equipment and staff is required to provide comprehensive veterinary healthcare coverage [31]. Some of our participants suggested fixed rotational visits to households, as these would enable them to plan their expenses and take preventive measures as advised by a professional. Simultaneously, trust and mutual understanding must be established in pastoral regions for people to cooperate with governmental veterinarians [30, 31]. For example, livestock professionals should acknowledge and build upon the broad knowledge farmers have of the conditions their animals suffer from and the types of treatment available to them [30]. In addition to rotational visits, seminars to guide farmers to recognise and handle given conditions, for example by implementing biosecurity and other preventative measures, may increase visibility of governmental services and help build rapport with farmers who often self-identify as experts on the treatment of their own livestock. As Caudell et al. [13] suggest, a bottom-up, rather than a top-down, approach holds promise, provided that interventions are co-designed with participants. Trust can then be established by demonstrating feasibility and success of interventions [13].

### AMR awareness and antibiotic stewardship training for improved prescribing and risk management

Patients and farmers often perceived antibiotics as similar to other medications, lacking an understanding of their specific purpose and accurate use. This was also demonstrated by a participant in Green et al.'s [21] study who described antibiotics as follows: “[Antibiotic] is the other word for medicine used to treat disease… I think there is no difference [between antibiotics and other drugs]”. The practice of crossover-use, i.e. the use of human medicines for animals and vice-versa, described in other studies in East Africa [38, 53], further substantiates that people do not see these drugs as different from each other. Such practices were often spread by word of mouth through informal networks. Therefore, relying on such incorrect advice, experience or old prescriptions can result in a treatment that may work but is not the most effective or safe [21, 31] and which can contribute to the development and spread of AMR [5]. Targeted awareness creation is needed to educate patients and farmers about the differences between treatment types, the impact of suboptimal antibiotic use on AMR, and the importance of demanding better quality of care and appropriate counselling.

However, medical professionals have been shown to also contribute to suboptimal antibiotic use. They may overprescribe antibiotics or provide outdated advice [5] due to diagnostic uncertainty [18, 23] or perceived patient pressure [31, 51, 54]. This highlights the need for ongoing training on antibiotic stewardship and AMR aimed at prescribers and dispensers. Hospital-based antibiotic providers in Tanzania have emphasised the importance of continued training to improve their practices [18], and health workers have expressed their motivation to support their community and enhance their services [15]. Tailored training for both public and private drug dispensers can improve decision-making around treatment, enhance provider–client communication, and further knowledge on AMR [55]. By developing providers' skills in antibiotic stewardship and AMR, accurate information on appropriate antimicrobial use can indirectly reach users. However, it is important to address underlying obstacles to the delivery of high-quality healthcare services to ensure that knowledge and awareness translate into meaningful changes in healthcare practices [15, 32] which will ultimately lower the risk of AMR development and spread [5].

### The role of the retail sector in human and veterinary health services

In the meantime, retail health providers in both the human and veterinary sectors should be more involved in overall health planning, cost allocations and training. Moreover, to make healthcare more affordable, including selected drug shops and pharmacies in insurance schemes would prevent double spending for insurance holders. Making affordable insurance schemes widely available and educating the public on how they can be used could increase the number of insurance holders in rural areas and encourage attendance of public health facilities. This would ideally lead to more prescriptions and advice from medical professionals for patients who still need to purchase their medicines from pharmacies. A study conducted in Moshi (northern Tanzania) showed that not having health insurance was associated with higher odds of suboptimal antibiotic use [25]. Similarly, the study by Mabilika et al. [14] in the Dodoma Region of Tanzania demonstrated an association between having health insurance and lower odds of self-treatment.

Mechanisms to involve the retail sector in antibiotic stewardship should also be considered, although this would require more flexible legislative frameworks to enable proper official training of human and animal retailers. This could involve granting approval for certain retail stores to directly sell quality-approved, first-line antibiotics to users. Currently, part 2 pharmacies can sell drugs on the Tanzanian list of essential medicines with a prescription, and agrovet stores can stock antibiotics to sell to veterinary professionals. However, these regulations are not effective in circumstances where individuals are unable to obtain a prescription and shops rely on over-the-counter sales for revenue. We recommend adaptation of the ABCD classification, taking into account regional disease patterns, to identify antibiotics that are crucial for Tanzanian farmers, to then enable over-the-counter sales of Access (human) and class D (animal) antibiotics to increase access to medicines critical for improving the health and productivity of livestock. Increased access could then be accompanied by the provision of guidance to sellers on the drugs’ correct usage and the type of advice clients should receive when buying them. This approach provides better control over antibiotics sold by private outlets, and the ability to restrict the sale of Watch and Reserve or class C, class B and class A antibiotics more stringently, without limiting access to healthcare for many. The eventual phasing out of over-the-counter sales should be considered only following increased accessibility through the public health system. For example, in South Africa, efforts have been made to render antibiotics available in all public human health facilities [56, 57]. As a result, self-treatment in this region is much lower than in Tanzania [58].

### The importance of interdisciplinary studies and One Health approaches for tackling self-treatment

Interdisciplinary studies which span various qualitative and quantitative methods are well suited to give insight into complex problems that require an in-depth understanding of perceptions, beliefs and practices as well as their drivers and causes. This understanding is critical to the design of interventions that are sustainable, and to devise solutions that are practical and viable for the target population [13, 59]. Drivers of self-treatment span a wide range of interacting structural, economic, political and social factors [32], all of which need to be addressed to reduce the occurrence of this problem [59]. Tailoring future programmes to locally relevant problems, circumstances and cultures is important to increase people’s sustained interest and engagement [60] and to foster trust in populations who historically lacked governmental support. To achieve this, bottom-up approaches via co-design with communities are needed [13, 32]. Therefore, involvement of social sciences, known for their participatory and collaborative nature, alongside other biomedical disciplines is essential [29, 61].

Furthermore, a One Health approach is necessary to bring stakeholders across various health sectors together and to overcome siloed thinking and approaches to address pervasive issues such as self-treatment. These issues impact both sectors in similar and interconnected ways, highlighting the need to find integrated solutions that consider these interactions and mutual challenges.

### Limitations of this study

Our study has some limitations that should be considered when interpreting the results.

Firstly, by carefully selecting localities with differential access to healthcare facilities and services, we aimed to capture the diversity and characteristics of communities within the region. While acknowledging that variations in healthcare access may exist across the larger region, the comprehensive nature of our research design and the representative nature of the chosen locations strengthen the validity and relevance of our findings to reflect the reality in northern Tanzania and across the region. Nevertheless, while our study included both formal and informal retailing, we collected very limited data from open markets and general stores which were exclusively included in the exit survey. We acknowledge the importance of the informal retail market in antibiotic provision in communities. Further investigation is warranted to fully comprehend its role in the provision of antibiotics and contribution to their subsequent use for human and livestock treatment.

Secondly, the three main datasets (drug bag, exit and provider surveys) relied on self-reported data from participants, which may be subject to desirability and recall bias. To mitigate this, we conducted the exit survey immediately after participants purchased their medicines, provided antibiotic samples to help with recall during the drug bag interviews and asked about antibiotics participants were most familiar with, i.e. the ones they had used most. We also acquired observational data to counterbalance desirability bias. However, the presence of researchers in the drug dispensing outlets may have altered the providers and/or clients' behaviours. Nevertheless, as the research team had been working with the study communities for a long time, building rapport and gaining trust, we do not believe that our presence compromised the quality of data in a meaningful way. We regularly observed practices which are deemed illegal (i.e. over-the-counter sales of antibiotics without a prescription) and felt that both users and providers talked openly about their experiences and problems with us.

Thirdly, we relied on translations from Swahili to English, which may have led to the loss of nuances and details in qualitative data. To address this, we referred back to the original Swahili transcript and consulted with field team members to ensure accuracy.

### Conclusions

Our research highlights the widespread occurrence of self-medication among farmers and patients in diverse agro-ecological production systems attributed to deficiencies in health care and public health system. Addressing the complex issue of self-treatment with antibiotics requires a comprehensive, interdisciplinary approach at multiple levels. Our research suggests that tackling systemic shortcomings of the public health system and improving the quality of health services at the point of care for patients and farmers are critical components of any successful intervention. A One Health framework is essential, involving diverse stakeholders including, but not limited to government agencies, local human and animal health and agricultural officials and end-users. It is important to recognise the right to healthcare and livelihoods of affected communities and work with retail providers, who are already a vital source of health information and products, to build a more resilient, accessible and high-quality public health system that can be trusted. Finally, by optimising awareness campaigns and improving access to good quality healthcare, we can hope to decrease the occurence of often suboptimal self-treatment, curbing the risks of AMR development while also ensuring a healthier future for all.

## Data Availability

The datasets generated for this study will be available from the corresponding author in anonymised form upon reasonable request. Survey forms are all available here: https://researchdata.gla.ac.uk/id/eprint/1561.
